# Gaps and barriers preventing sustained delivery of HPV vaccination in Kano, Kaduna, and Lagos states: An exploratory qualitative study

**DOI:** 10.1371/journal.pone.0350510

**Published:** 2026-06-18

**Authors:** Chidozie Ezechukwu, Esther Oluwayemisi Ayandipo, Demilade Osoteku, Raihanah Ibrahim, Oluwafunmilayo Raheem, Amy Boldosser-Boesch, Sarah Birse, Elizabeth Weinstein, Justice Nonvignon, Goodness Hadley, Eric Aigbogun, Uchenna Igbokwe

**Affiliations:** 1 Management Sciences for Health (MSH), Abuja, Federal Capital Territory, Nigeria; 2 Solina Centre for International Development and Research (SCIDaR), Abuja, Federal Capital Territory, Nigeria; 3 Management Sciences for Health (MSH), Arlington, Virginia, United States of America; 4 Women Advocates for Vaccine Access (WAVA), Abuja, Federal Capital Territory, Nigeria; FHI 360, ZAMBIA

## Abstract

**Background:**

To lessen the burden of cervical cancer, Nigeria introduced the HPV vaccine to its national immunization program, but the program has faced operational and contextual problems during its early rollout. This study explored the systemic and community-level barriers affecting HPV vaccine delivery across selected Nigerian states.

**Method:**

This exploratory qualitative study employed key informant interviews with policymakers, immunization officers, government agencies, community-based organizations, and implementing partners across three states: Kaduna, Kano, and Lagos, from April 2025 to October 2025. Data were thematically analyzed using NVivo, with emphasis on identifying factors influencing the sustainability of HPV vaccination. The analysis focused on key domains such as policy implementation, financing, service delivery, supply chain systems, human resources, data management, and demand generation.

**Result:**

The study found that HPV vaccine implementation is hindered by limited state-level policy adaptation, reliance on donor funding, human resource shortages, supply chain weaknesses, data management gaps, and strong socio-cultural resistance driven by misinformation. These interconnected barriers reduce vaccine acceptance and disrupt service delivery.

**Conclusion:**

The HPV vaccination program in Nigeria is constrained by systemic weaknesses and socio-cultural resistance. Addressing these challenges requires stronger state-level policy adaptation, sustainable financing, improved supply chain and data systems, and targeted community engagement to enhance acceptance and ensure effective service delivery.

## Background

Human papillomavirus (HPV) is one of the most common sexually transmitted infections globally, and is a well-established cause of cervical cancer, as well as other anal, vulvar, and oropharyngeal cancers) [1,2]. The causal relationship between HPV and cervical cancer was first proposed by zur Hausen in the 1970s, leading to the identification of high-risk HPV genotypes 16 and 18 as the primary oncogenic strains responsible for approximately 70% of cervical cancer cases [3]. This discovery laid the foundation for HPV testing and the development of prophylactic vaccines, transforming cervical cancer prevention into a vaccine-preventable strategy aligned with the World Health Organization’s (WHO) cervical cancer elimination goals [4,5]. Beyond cervical cancer, HPV is also associated with other malignancies, including anal, vulvar, and oropharyngeal cancers [6].

Globally, cervical cancer ranks as the fourth most common cancer among women, with an estimated 660,000 new cases and 350,000 deaths reported in 2022 [7]. More than 80% of these cases occur in low- and middle-income countries (LMICs), where access to screening and preventive services remains limited [8]. Sub-Saharan Africa bears a disproportionate share of this burden, with the highest reported HPV prevalence globally, estimated at approximately 22.1%, although prevalence varies widely depending on study population and geographic context [9,10].

In Nigeria, HPV prevalence varies by region, population, and study year, with reported estimates ranging from approximately 12% to over 40% in some populations [11–13]. Cervical cancer is the second most frequent cancer among women in Nigeria and the leading cause of mortality and morbidity among women aged 15–44 years, with approximately 12,000 new cases and 8,000 deaths reported annually [14–16]. Despite this high burden, HPV vaccine uptake remains low. In a 2024 study in Northern Nigeria, [17]found that even if the HPV vaccine were free or subsidized, approximately one-third (32.7%) of parents would still choose not to vaccinate their children against the virus, highlighting a substantial gap between the implementation of national vaccination policies and actual population-level uptake [18,19].

In November 2023, Nigeria introduced the HPV vaccine into its routine immunization programme, targeting approximately 7.7 million girls aged 9–14 years in the largest HPV vaccination rollout in Africa [20], with the aim of contributing to the global 90-70-90 target (90% of girls fully vaccinated by age 15, 70% of women screened by ages 35 and 45, and 90% of women with cervical disease treated) for cervical cancer elimination by 2030 [17]. The vaccine is most effective when administered prior to sexual debut, making early adolescence a critical period for intervention [21]. However, early implementation experiences suggest that the scale-up of HPV vaccination in Nigeria is constrained by multiple challenges, including logistical and supply chain weaknesses, inadequate human resources, weak data systems, and persistent vaccine hesitancy driven by misinformation and sociocultural beliefs [22,23]. Evidence from Nigeria and other LMICs indicates that successful HPV vaccine delivery depends not only on national policy adoption but also on effective subnational implementation [24,25].

Nigeria operates a three-tier, decentralized health system, comprising of the federal government, who provides policy direction, regulation, and tertiary services; state governments who are responsible for secondary services and implementation of national policies and strategies; and local government who are responsible for primary health care delivery [26,27]. This system places substantial responsibility for financing, service delivery, data management, and community engagement on state and local governments. Weaknesses across these domains, combined with sociocultural resistance, limited community awareness, and inconsistent demand generation, threaten the sustainability and equity of HPV vaccination programmes [28,29]. A particularly salient systemic constraint is decentralized implementation without commensurate fiscal responsibility at sub-national levels [30]. Operationally, states coordinate and implement health programs; however, many of them remain heavily dependent on external donor financing due to weak budgetary commitment to immunization programmes, resulting in an unfunded mandate that undermines sustainable ownership and long-term scalability [31].

To systematically examine these constraints with regards to ` HPV vaccination in the country, this study appraises the WHO health systems building blocks framework, which conceptualizes health systems across six components: leadership and governance, health financing, health workforce, medical products and technologies, health information systems, and service delivery. This analytical lens enables a structured assessment of how system-level bottlenecks shape implementation performance. While previous studies have examined HPV vaccine acceptability, caregiver knowledge, and individual-level determinants of uptake in Nigeria [8,14], there remains limited qualitative evidence exploring the systemic, programmatic, and societal barriers affecting sustained HPV vaccine delivery across diverse state contexts. Addressing this evidence gap is essential to informing strategies that strengthen implementation, improve coverage, and support Nigeria’s progress toward cervical cancer elimination. Against this background, this study explores the gaps and barriers influencing HPV vaccination delivery in Kano, Kaduna, and Lagos states, with the aim of generating actionable insights to support more effective and equitable implementation of Nigeria’s HPV vaccination program.

## Materials and methods

### Study design

The study employed an exploratory qualitative research design using key informant interviews (KIIs) and focus group discussions (FGDs) to explore gaps and barriers preventing sustained delivery of HPV vaccination in Kaduna, Kano, and Lagos states, Nigeria. Data collection was conducted between April 2025 and October 2025 among key stakeholders involved in HPV vaccination implementation, and data transcription and analysis were completed following the end of data collection.

### Study conceptual and exploratory framework

**Fig 1:**
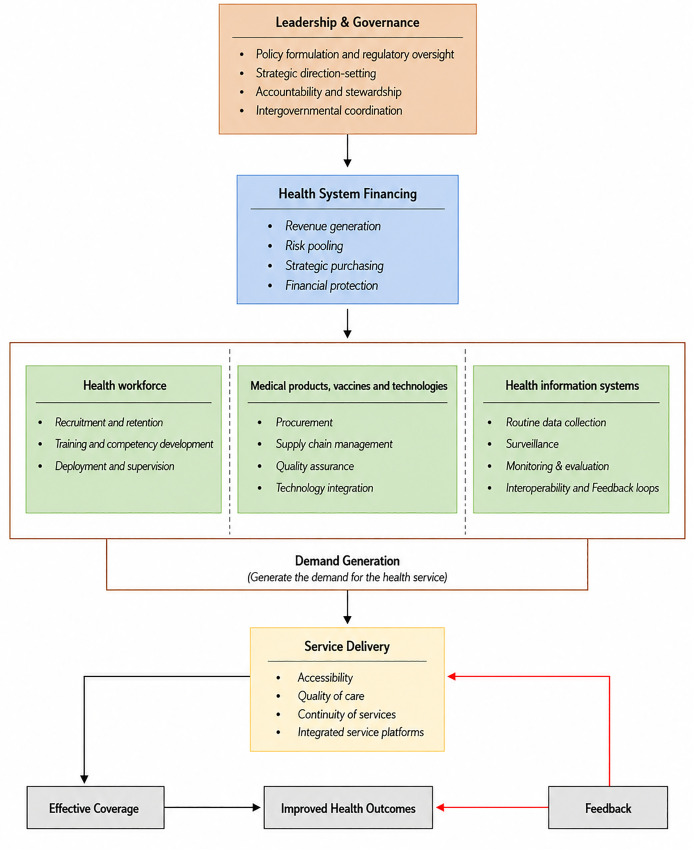
Study conceptual and exploratory framework. Adapted from the WHO Health Systems Building Blocks (WHO, 2010).

This study was guided by the WHO Health Systems Building Blocks framework, which describes health systems in 6 core interdependent components: leadership and governance, health financing, health workforce, vaccines and technologies, health information systems, and service delivery [32]. The study framework (Fig 1) assumes that governance and financing create the enabling environment through policy formulation and regulatory oversight, strategic direction-setting, and accountability and stewardship, Revenue generation, risk pooling, strategic purchasing, and providing financial protection. This action is intended to guarantee adequacy of workforce (human resource for health; HRH), commodities, and information systems, which ensures operational readiness and performance. The availability of the 3 critical components should guarantee quality service delivery that translate into effective vaccination coverage. Improved coverage drives the country to achieving the 90-70-90 goal and indication for better health outcomes. The collection and use of routine data enables feedback loops that inform adaptive policy and implementation adjustments for efficiency in service delivery and better health outcomes.

### Participant sampling and recruitment

The study employed purposive sampling to recruit key stakeholders involved in the planning, financing, delivery, and community engagement components of HPV vaccination across Kaduna, Kano, and Lagos states [33]. Eligible participants were individuals directly engaged in HPV vaccination implementation at state, local government, or community levels including policymakers, immunization officers, implementing partners, government agencies, community-based organizations (CBOs), and non-governmental organizations (NGOs).

Participants were identified during initial state-level engagements conducted as part of HPV vaccination programme activities in each state. These engagements facilitated the identification of individuals with relevant technical, operational, and community-level experience. Potential participants were approached directly and invited to participate in either key informant interviews (KIIs) and focus group discussions (FGDs) based on their roles, expertise, and availability.

A total of 55 stakeholders participated in the study across Kaduna (n = 19), Kano (n = 14), and Lagos (n = 22) States (breakdown as seen in Table 1). Participants were recruited through purposive sampling to ensure representation of key actors involved in HPV vaccine implementation. Thirty-eight (38) KIIs were conducted with policymakers (n = 6), immunization programme managers (n = 3), CBO/NGO representatives (n = 9), government agency representatives (n = 8), and implementing partners (n = 12). In addition, three FGDs were conducted with immunization officers, comprising 6 participants in Kaduna, 4 in Kano and 7 participants in Lagos (n = 17). The inclusion of stakeholders from multiple implementation levels enabled exploration of a broad range of experiences and perspectives related to HPV vaccine delivery. Data collection proceeded iteratively and was concluded when sufficient information power was achieved across stakeholder categories and study settings.

**Table 1:** Breakdown of participants by stakeholder categories.

S/N	Summary	Kaduna	Kano	Lagos
**1**	**Policymakers**	**2**	2	2
**2**	**Immunization Program Managers**	**1**	1	1
**3**	**CBOs/NGOs**	**3**	2	4
**4**	**Government & Agencies**	**3**	2	3
**5**	**Implementing Partners**	**4**	3	5
**6**	**FGD Participants**	**6**	4	7
**Total**		**19**	14	22

### Study setting and context

The study was conducted in Kaduna, Kano, and Lagos states of Nigeria. These states were purposively selected to reflect geographic, socio-cultural, and health system diversity relevant to HPV vaccine implementation across Nigeria. Kaduna and Kano are located in the northwestern region, while Lagos is situated in the Southwestern region of the country, allowing exploration of contextual differences across regions with varying demographic profiles, health service delivery structures, and sociocultural norms.

Kaduna State is one of Nigeria’s largest states, comprising 23 local government areas (LGAs), with a mix of urban and rural communities. Kano State is one of the most populous states in Nigeria and has experienced rapid urban growth, which presents both opportunities and challenges for large-scale immunization delivery [34,35].

Lagos State is Nigeria’s most populous and urbanized state, with an estimated population of approximately 26 million as of 2019 and high population mobility [36]. Projections suggest continued population expansion, further increasing pressure on health service delivery systems [37]. Lagos also has a high concentration of health facilities, including routine immunization centres, providing a contrasting implementation context to predominantly northern settings.

These three states were also selected because they were actively implementing HPV vaccination during the study period, providing an opportunity to examine operational, systemic, and community-level factors influencing HPV vaccine delivery across diverse implementation contexts.

### Data collection procedure

Data collection was conducted using key informant interviews (KIIs) and focus group discussions (FGDs) to obtain in-depth perspectives from stakeholders involved in HPV vaccination implementation across Kaduna, Kano, and Lagos states. Data collection took place between April 2025 and October 2025 and was conducted by trained members of the research team with experience in qualitative data collection. Participant recruitment commenced on 16/04/2025 and ended on 03/06/2025.

Semi-structured interview guides were developed to guide both KIIs and FGDs (Appendix II). The guides were structured around seven thematic areas relevant to HPV vaccine implementation: policy and strategic planning, financing and financial management, vaccine and supply chain management, service delivery, demand generation and mobilization, data management and use for decision-making, and human resources for health. The guides were used flexibly to allow participants to elaborate on issues most relevant to their roles and experiences.

KIIs were conducted with individuals in leadership, policy, or technical roles who possessed in-depth knowledge of HPV vaccination planning and implementation. Focus group discussions were conducted with participants whose roles involved operational or community-level engagement, enabling shared reflection on implementation experiences and challenges.

All interviews and FGDs were audio-recorded with participants’ consent and supported by debrief notes taken by the research team. These debrief notes informed preliminary reflections and were used alongside interview transcripts during analysis.

Data collection continued until thematic saturation was reached, defined as the point at which no new codes or themes emerged from successive KIIs and FGDs.

### Data management and analysis

All the audio-recordings from the KIIs and FGDs were transcribed verbatim, and cleaned to ensure accuracy and completeness, which was managed on a roll-in basis to ensure thematic saturation. Saturation was assessed iteratively by the research team using a Miro board after every five KIIs and the FGDs, with a final review confirming that the last two KIIs from each state did not produce any new information relevant to the study objectives. As an initial step, the research team conducted a rapid review of debrief notes on the Miro board to capture emerging issues and early insights, which informed preliminary thematic organization.

Codebook was developed deductively based on the seven thematic domains (policy and strategic planning, financing and financial management, vaccine and supply chain management, service delivery, demand generation and mobilization, data management and use, and human resources for health) derived from the interview guides. Four researchers (EOA, EA, OR, GH) independently coded the first three transcripts (one per state) to ensure consistency in code application (grounded theory approach). The team then convened to compare outputs, resolve discrepancies through consensus, and refine the codebook. Subsequently, all remaining transcripts were double-coded by rotating pairs from the research team (EOA, EA, OR, GH), with disagreements resolved by a third reviewer (RI). The team held regular team debriefings to maintain coding consistency and address emerging analytical issues. While inter-coder reliability was not quantified formally, the study applied a consensus-based approach throughout the process to ensure alignment. NVivo 14 software [38]was used to manage the coding process and facilitate theme development.

Following coding, a team-based inductive thematic analysis was conducted. This involved iterative reading of transcripts to identify patterns, relationships, and recurring ideas within and across the predefined thematic domains. Affinity diagramming using Miro board (an online collaborative whiteboarding platform or visual workspace for innovation) was used to cluster related codes into higher-order categories and refine overarching themes, ensuring that findings captured diverse perspectives across stakeholder groups.

Comparative analysis was conducted across states (Kaduna, Kano, and Lagos) and stakeholder categories to identify convergences and contextual variations. Key discrepancies and unique findings were further examined during validation workshops with stakeholders, which served to verify interpretations, enhance credibility, and generate additional insights incorporated into the final analysis.

### Validation workshops

Validation workshops were conducted in Lagos, Kano, and Kaduna states following preliminary data analysis to present emerging findings, clarify interpretations, and generate additional perspectives that may not have been captured during the key informant interviews and focus group discussions.

The workshops included presentations of preliminary findings, breakout group discussions, and plenary feedback sessions with participating stakeholders. These sessions facilitated active stakeholder engagement and provided an opportunity to reflect on similarities and differences across states and stakeholder groups.

Feedback from the validation workshops was used to verify findings, clarify contextual nuances, and refine thematic interpretations, thereby strengthening the credibility and trustworthiness of the study results.

### Ethical considerations

The study was conducted in accordance with the ethical principles outlined in the Declaration of Helsinki for research involving human participants. Ethical approval was obtained from the National Health Research Ethics Committee of Nigeria (NHREC), which reviewed and approved the study protocol and informed consent forms provided in English. The NHREC approval number is NHREC/01/01/2007-04/04/2025. The study was conducted in strict compliance with ethical standards for research involving human participants, including data collection.

All stakeholders were provided with adequate information about the study prior to giving written informed consent. Respondents were required to sign a consent form explicitly indicating their agreement to participate in key informant interviews and focus group discussions, as well as the use of insights for dissemination to relevant stakeholders. Participation was voluntary, and stakeholders were informed that they could withdraw at any time without repercussion. The study included only adult participants, and no financial incentives were provided.

Interview and focus group discussion transcripts were anonymized using codes indicating state and respondent type (e.g., IP, PM), and all data were stored securely with access restricted to authorized members of the research team. These measures ensured that the rights, privacy, and dignity of participants were respected throughout the study.

## Result

The result from the affinity diagramming reflected systemic barriers across the different aspects of the HPV vaccination efforts.

### Policy and strategic planning

In the area of policy and strategic planning, the analysis revealed fragmented frameworks that often led to inconsistent implementation. The adoption of the national strategic document for HPV vaccination was not contextualized to the state realities, and the mechanism for ensuring programmatic accountability remained weak, resulting in difficulty in translating policy into effective action (Table 2).

**Table 2:** Findings from affinity mapping by thematic areas and states.

Theme	Kaduna State	Kano State	Lagos State
**Policy and Strategic Planning**	Lack of state-specific policies; weak health worker capacity; limited community engagement; gaps in vaccination, eligibility, and treatment guidelines hinder implementation.	No state-specific HPV policy; relies on national policy, limiting effective adaptation and implementation at state level.	HPV vaccination limited to girls aged 9–14 and not embedded in broader health policies; reliance on partner funding and weak routine policy support affects school-based delivery.
**Financing and Financial Management**	Limited government funding and weak resource mobilization, with heavy reliance on partners for HPV program financing.	Dependence on routine immunization budgets and partners; no dedicated funding, delayed disbursements, unclear fund flows, and limited innovative financing.	Heavy reliance on external partners with no dedicated government funding, raising sustainability concerns; limited resources constrain coverage of target age group (9–14 years).
**Vaccine Supply Chain Management**	Stock-outs and unequal distribution; high-demand areas experience shortages while low-demand areas have unused vaccines.	Frequent stock-outs, incomplete kits, weak cold chain coordination, lack of dedicated funding, and poor follow-up systems affecting delivery and tracking.	Unpredictable demand, unrealistic targets, and logistical inconsistencies; one-dose vials strain LGA-level cold chain capacity, especially in hard-to-reach areas.
**Human Resources for Health**	Severe workforce shortages, low knowledge levels, reliance on unmotivated volunteers, compounded by insecurity and burnout.	Rural facilities often have only one staff member; shortages due to retirements and low recruitment; limited structured HPV training and weak supervision.	Overstretched RI officers, limited training time, and inexperienced new staff; persistent workforce shortages driven by attrition and slow recruitment.
**Data Management & Decision Making**	Lack of standard registers, reporting discrepancies, limited staff capacity, and weak DHIS integration despite data flow for decision-making.	DHIS2 downtime, SMS failures, poor connectivity, and weak digital systems; incomplete reporting from hard-to-reach areas and inconsistent data quality.	Manual reporting, delayed submissions, weak private sector compliance, lack of digital tools, and poor feedback systems limiting data quality and timeliness.
**Demand Generation & Social Mobilization**	Misinformation, cultural stigma, male influence, religious myths (infertility), and insecurity hinder vaccine uptake in hard-to-reach areas.	Widespread misinformation (infertility, promiscuity), male-dominated decisions, and religious sensitivities persist despite some traditional leader engagement.	COVID-era myths, fertility concerns, teacher misinformation, religious conservatism, and paternal consent refusal drive hesitancy and reduce uptake.

Findings across the three states revealed an absence of state-specific HPV vaccination policies, with all states relying exclusively on national guidelines. One of the policymakers (PM) explained:

*“We do not actually have a state-specific policy. We are riding on the existing national policies… routine immunisation from birth to toddlers, and now the introduction of HPV vaccination.”*

Participants also identified gaps within the national policy that may affect the long-term sustainability of the HPV programme. A key concern was that HPV vaccination targets girls aged 9–14 years, unlike routine immunisation, which focuses on children under two years who are easier to reach through established facility-based services and are not gender-specific. Targeting older girls presents distinct challenges, as many are in school and require both parental consent and school approval for vaccination. Although the Federal Government, in collaboration with the NPHCDA and NICRAT, has integrated HPV vaccination into the school health programme [23], implementation barriers persist. Some parents refuse vaccination, perceiving their daughters as too young or “fragile” for a vaccine associated with a sexually transmitted infection. The exclusion of boys from the programme was also viewed as potentially fuelling suspicion. Additionally, concerns were raised about vaccinating girls who may already be sexually active. One participant from the implementers noted:

*“Unlike the routine vaccines We are used to, which are normally given to children below the age of two, the HPV vaccine targets older age groups not in the RI age group.”*

Another policymaker highlighted additional policy-related challenges:

*“One of the major gaps was the issue of the age range. Then also, another gap was that teachers themselves didn’t understand what the vaccine was all about.”*

Some policymakers suggested that revising national guidelines to include boys could help increase acceptance and uptake of the vaccine. As one participant stated:

*“Maybe if we expand the population a bit, maybe if we include other people, like boys, that suspicious thing will hopefully fizzle out… Community engagement could have done better.”*

However, not all participants perceived these issues as policy gaps. One policymaker emphasized that the challenges were primarily implementation-related:

*“It’s more of an implementation issue, not a policy gap. A lot of work has to be done around community awareness, mobilization, and advocacy to high-level personalities to bring everyone on board.”*

### Financing and financial management

The affinity mapping highlighted that the budget allocation for HPV vaccination was rather insufficient, with significant delays in fund disbursement and weak accountability mechanisms. These issues disrupt program implementation and limit the ability to plan and implement long-term strategies. There was also the concern about sustainability associated with heavy reliance on donor funding (Table 2).

Across all three states, financing for HPV vaccination activities including community awareness and service delivery is largely supported by implementing partners and the National Primary Health Care Development Agency (NPHCDA), with minimal direct state government funding.

An immunization officer explained that funding for the programme is currently driven by external support:

*“A large portion of the funding for HPV vaccination comes from partner support. Funding operates under an ‘envelope system’ at the primary health care board.”*

Similarly, a policymaker emphasized the reliance on development partners, noting that state contributions mainly cover logistics and personnel:

*“We depend a lot on international partners… development partners. The state also partly funds implementation by providing logistical support and human resources.”*

A major financing gap identified across states is the absence of a dedicated budget line for HPV vaccination. As one policymaker stated:

*“There is no specific, dedicated budget line for routine immunization, which includes HPV vaccination, in the state’s budget.”*

This dependence on donors is further illustrated by another respondent who explained that states often rely on partner-driven outreach:

*“We are leveraging for outreach by other partners. When they come, they come and support us.”*

Delayed fund disbursement and unclear financial flows were also highlighted as challenges that weaken financial management and compromise effective programme implementation.

Given these gaps, stakeholders recommended the establishment of dedicated HPV budget lines within state budgets, increased government financial commitment to reduce donor dependence, and strengthened financial transparency, combined with timely disbursement mechanisms to support sustainability.

### Vaccine supply chain management

From the affinity mapping (Table 2), the issues with the vaccine supply chain management stemmed from frequent stockouts due to poor forecasting and distribution. Inadequate storage facilities at local levels and logistical challenges associated with the last-mile delivery directly affected vaccination efforts at target locations.

Vaccine delivery across the three states is constrained by multiple supply chain challenges, including incomplete vaccine kits, frequent stock-outs in high-demand locations, and inadequate cold chain capacity. These issues are compounded by poor coordination and unpredictable demand patterns, which limit the efficiency of vaccine distribution. An implementing partner described the inequitable distribution:

*“Yes… supply is not equitable. In areas with high demand, they run out, while in areas without demand creation, vaccines there are unused.”*

Stakeholders emphasized the need for stronger logistics systems to ensure consistent vaccine availability.

As one implementing partner explained:

*“Accurate data collection and microplanning, strengthening monitoring and evaluation, logistics and financial support, and improved technology and capacity building for data management.”*

Key recommendations included improving cold chain infrastructure, enhancing microplanning, and adopting real-time stock monitoring systems. Consistent funding for supply chain activities was also highlighted as essential for uninterrupted vaccine delivery.

### Human resources for health

From the analysis, stakeholders noted shortages of trained personnel, particularly at the community level. There was also the issue of unequitable distribution of health workers across regions, with inadequate incentives for healthcare workers’ retention (Table 2).

Across all three states, respondents consistently reported significant shortages of human resources for health, particularly in rural areas where a single health worker is often responsible for delivering multiple services. A CBO representative emphasized the extent of the gap:

“Human resources is a major problem. We don’t have enough trained personnel on HPV.”

The HPV vaccination programme also relies heavily on temporary or ad hoc staff, many of whom are poorly compensated, insufficiently trained, and frequently overworked. This situation contributes to burnout and low motivation among frontline workers. An implementing partner highlighted these challenges:

“Many health workers are temporary staff who are not paid living wages. They are overworked, leading to pockets of burnout and low motivation.”

Participants further noted the absence of structured HPV-specific training or refresher courses, which limits the capacity of health workers to deliver high-quality services.

To address these gaps, stakeholders recommended recruiting and retaining more skilled health workers in underserved areas, institutionalizing regular HPV-focused training and refresher programmes, and introducing incentives to improve the motivation and performance of temporary staff.

### Data management and data for decision making

From the affinity mapping (Table 2), data management and decision-making emerged as another significant barrier. While the systems both state-specific or national were optimized to provide real-time and reliable data for routine immunisations, there was no adaptability for HPV vaccination campaigns, which hindered the ability to guide interventions effectively. There was also limited capacity for analysis and use in planning, which in turn weakens the evidence base for decision-making and reduces the responsiveness of programs.

Across the three states, District Health Information System2 (DHIS2) serves as the primary platform for data management, supported by local government–level registers, tally sheets, and Open Data Kit (ODK) tools. These data are eventually uploaded into DHIS2. However, the system faces persistent operational challenges, including server downtime, poor network connectivity, unreliable power supply, and the absence of digital data entry systems in many rural LGAs. Data discrepancies were particularly noted as a significant issue in Kaduna State. An implementing partner explained:

“Yes… data discrepancies, number of doses not tallying with girls vaccinated. Some facilities do not have that accountability to give you the data that you want.”

Another challenge is the incomplete integration of HPV indicators into routine immunization reporting tools. An immunization officer illustrated this gap:

“Previously, standard registers, tally sheets, and summaries did not have dedicated columns for HPV vaccination. Healthcare workers improvised by drawing a margin line and writing ‘HPV’ in the comment section.”

Stakeholders emphasized the need to strengthen monitoring and evaluation systems, fully integrate HPV indicators into routine immunization tools, provide digital devices and data management training for health workers, and improve infrastructure for data reporting, particularly in rural areas.

### Demand generation and social mobilization

Demand generation and social mobilisation were also constrained by poor community engagement and trust-building. This was compounded by cultural and religious beliefs that antagonize HPV vaccination. There were also ineffective communication strategies that were not adequately tailored to the diverse populations in the three states. As a result, efforts to mobilize communities were often met with resistance or limited uptake (Table 2).

Persistent misinformation continues to undermine HPV vaccine uptake across the three states. Common misconceptions include claims that the vaccine causes infertility, is a form of secret family planning, or encourages promiscuity among young girls. One implementing partner highlighted these concerns:

*“Association of HPV with family planning, fear of sterilization, and belief that girls are too young for sexual health topics.”*

Another respondent noted that misinformation is particularly prevalent in northern Nigeria, where distrust of vaccines remains high:

*“In the Northern part of Nigeria… misconceptions about vaccine safety and effectiveness. COVID-19 vaccine rumours may also affect HPV uptake. The use of gestures and influencers is helpful.”*

Cultural and religious resistance, combined with irregular and unsustained awareness campaigns, further weakens demand for the vaccine. A CBO representative explained:

“Some communities resist due to traditional beliefs or religious reasons. Some believe there’s no need because they’ve ‘always been fine.’”

Participants emphasized that trusted community messengers such as religious leaders, traditional rulers, teachers, and civil society organizations play a central role in shaping community perceptions. As one respondent stated:

*“They play a major role… we should start from them. Pastors and community influencers can shift perceptions and drive uptake.”*

To address these demand-side barriers, stakeholders recommended leveraging influential community leaders, delivering consistent and culturally appropriate awareness campaigns in local languages, and sharing survivor stories to counter misinformation, build trust, and strengthen vaccine acceptance.

### Service delivery

The affinity mapping showed that the quality of service delivery was inconsistent, particularly with the outreach sessions that targeted the hard-to-reach communities. There were also infrastructure challenges that affected cold chain maintenance, which further compromised the reliability of services (Table 2).

Although HPV vaccination has been integrated into routine immunization services, delivery remains constrained by multiple operational and community-level barriers. Key challenges include shortages of human resources for health (HRH), limited outreach capacity, and persistent socio-cultural resistance. Participants explained that outreach teams are often unable to reach underserved communities, and service access is further restricted by costs in private health facilities.

Several barriers were identified at health facilities, schools, and within communities, including rumours linking the vaccine to infertility, resistance from school authorities and parents, security concerns, and health worker shortages. One community-based organization (CBO) representative highlighted the impact of misinformation:

*“The barriers we majorly face are from the teachers, LGAs, and communities. Rumours that the HPV vaccine causes infertility.”*

Another CBO respondent described additional social and structural barriers:

*“There are cultural and social barriers… It starts with churches and mosques, then traditional leaders. Also, because of the JAPA syndrome, healthcare workers are overwhelmed… limited access in rural areas… private facilities aren’t involved enough.”*

Participants also noted disparities in service delivery between urban and rural areas, highlighting the need for tailored strategies to reach hard-to-reach communities.

Overall, improving service delivery will require expanded targeted outreach to marginalized girls, strengthening the capacity of health workers for integrated service delivery, engaging school and community leadership structures, and addressing financial barriers particularly in private facilities to promote equitable access to HPV vaccination.

## Discussion

This study provides an in-depth understanding of the systemic and contextual barriers affecting HPV vaccine delivery across three Nigerian states. While national guidelines provide a framework for HPV vaccination, the findings demonstrate that implementation at state and community levels remains fragmented, under-resourced, and constrained by intersecting policy, operational, and socio-cultural challenges. Together, these barriers limit Nigeria’s ability to achieve high coverage among girls aged 9–14 years, despite global recommendations positioning HPV vaccination as an essential cervical cancer prevention strategy [20].

A central finding is the absence of state-specific HPV vaccination policies, resulting in heavy reliance on national guidance without sufficient adaptation to local contexts. The absence of state-specific policies disproportionately affects HPV vaccination compared to other routine immunizations (RI) for several interconnected reasons. First, routine immunizations target infants and toddlers (0–23 months), a population consistently reached through facility-based services and established maternal and child health platforms. In contrast, HPV vaccination targets girls aged 9–14 years, a group outside the traditional RI age range, requiring novel delivery strategies such as school-based campaigns and community outreach, as well as other non-convention outreach strategies modelled to the local context (like nomadic populations in the north and riverine dwellers in riverine communities). Without state-level policies to guide adolescent data capture, school engagement, parental consent procedures, implementation becomes ad hoc and inconsistent. Second, the exclusion of boys from the national HPV policy, as noted by participants, fuels community suspicion that the vaccine is linked to a ‘hidden agenda’ (for example, secret family planning or promoting promiscuity). Revising national guidelines to include boys could potentially reduce this stigma, as the vaccine would be perceived as a universal public health intervention rather than one targeting only girls. However, we understand that with limited funding and the interruption strategy, there is the need for better community engagement and advocacy strategy to tailor messaging to the context of the diverse communities within the state. Third, the absence of state-specific legislation creates a governance vacuum where accountability for adolescent vaccination is unclear, unlike routine immunization which benefits from decades of decentralized operational planning. Thus, while federal policy provides the mandate, the lack of subnational adaptation, including clear financing, target-setting, and implementation guidelines, renders HPV vaccination more vulnerable to operational failure and sociocultural resistance than the already established RI programs. Evidence from other LMICs underscores the importance of contextualizing national immunization strategies to subnational needs, particularly where school-based delivery and community mobilization are central to program success [24,39].

Financing constraints emerged as a major determinant of program sustainability across states. The study found that HPV vaccine delivery remains heavily dependent on donor and implementing partner support, with limited state-level financial ownership and the absence of dedicated budget lines. Similar patterns have been reported across sub-Saharan Africa, where donor-led HPV vaccination programs face challenges in sustaining activities such as logistics, training, community mobilization, and cold chain maintenance once external funding declines [25,29,40]. Delayed fund disbursement and unclear resource flows further weaken implementation and disproportionately affect outreach efforts in hard-to-reach communities.

Service delivery constraints were also prominent, particularly shortages of human resources for health, especially in rural areas. The reliance on temporary and inadequately trained health workers contributes to burnout, reduced motivation, and inconsistent service quality, limiting the capacity to conduct effective outreach and school-based vaccination. Vaccinators often do not know how to address the stiff resistance from school owners, teachers, parents, and guardians. Rural outreach gaps were also noted, as access and logistics hindered reach, and private facility participation was a major limitation, unlike routine infant immunization services, which are well institutionalized within primary healthcare facilities. These findings align with previous research demonstrating that the success of HPV vaccination programmes depends on adequately staffed and well-supported frontline workers, who play a crucial role in addressing caregiver concerns and facilitating school- and community-based delivery of the HPV vaccine [25,41,42].

Supply chain weaknesses further constrained effective HPV vaccine delivery. Participants described inequitable distribution, frequent stock-outs, incomplete vaccine kits, and inadequate cold chain capacity. These operational weaknesses were exacerbated by weak coordination and unpredictable demand patterns, leading to both wastage and shortages. Similar challenges have been documented in HPV vaccine introductions in Ethiopia, Malawi, and Uganda, where insufficient cold chain infrastructure and suboptimal microplanning resulted in stock-outs and disrupted vaccination schedules [43,44].

Data management limitations also affected programme performance. Although DHIS2 serves as the main data platform, system downtime, power and network challenges, and incomplete integration of HPV indicators into routine reporting tools undermine data reliability and use for decision-making. These findings reflect broader challenges within Nigeria’s immunization data systems, where poor data quality and accountability constrain evidence-based planning and monitoring [45,46].

Beyond system-level barriers, socio-cultural and religious factors strongly influence vaccine demand. Persistent misinformation, particularly rumours linking the HPV vaccine to infertility, secret contraception, or increased promiscuity, was reported across states and is consistent with global evidence identifying misconceptions as a key barrier to HPV vaccine uptake [8,47]. While religious and traditional leaders were recognized as influential actors, inconsistent and unsustained awareness campaigns limited their ability to support effective community mobilization.

The study also noted state-specific barriers. In Lagos, uptake was affected by elitism and hesitancy among highly educated urban populations, misinformation via social media, and a perceived lack of visible impact from vaccination efforts. Healthcare workforce shortages, poor staff-to-population ratios, and migrant populations also complicated planning and contributed to inadequate vaccine availability. In Kano, similar challenges related to high population density and mobility affected compliance and coverage, alongside language barriers and conspiracy theories framed around Western influence. Kaduna faced barriers including vaccine hesitancy in private schools and limited domestication of the national policy.

When considered holistically, these findings highlight the need for a comprehensive, multisectoral approach to strengthening HPV vaccine implementation in Nigeria. Strengthening state-level ownership through context-specific policies and dedicated financing mechanisms will be essential to reduce donor dependence and enhance sustainability. Improvements in service delivery will require investments in workforce capacity, structured training, supportive supervision, outreach microplanning, and cold chain systems. Addressing misinformation will depend on sustained engagement with trusted community actors, consistent culturally appropriate messaging, and locally resonant communication strategies.

Finally, strengthening data systems through full integration of HPV indicators into routine reporting, improved infrastructure, and digital technologies will be critical for responsive programme management and accountability. Collectively, these measures are essential to translate HPV vaccine introduction into equitable coverage gains.

Overall, the study demonstrates that while Nigeria has made progress in introducing the HPV vaccine, multiple systemic and sociocultural barriers continue to impede effective implementation. Addressing these gaps holistically will be essential for achieving high coverage, ensuring equitable access, and ultimately reducing the burden of cervical cancer in the country.

## Conclusion

This study highlights the multifaceted barriers affecting HPV vaccine implementation across three Nigerian states and reveals persistent gaps in community demand, data systems, human resources, supply chain management, service delivery, financing, and policy adaptation. While the introduction of the HPV vaccine represents a significant advancement in cervical cancer prevention, progress toward achieving high and equitable coverage remains constrained by a lack of state-level ownership, operational inefficiencies, and widespread socio-cultural opposition.

Strengthening subnational governance systems, securing sustainable domestic financing, and investing in health workforce capacity are necessary steps to enhance programme delivery. Equally important is the need for culturally responsive and sustained community engagement, alongside robust data systems that support timely monitoring and evidence-based decision-making.

Addressing these interconnected systemic and contextual barriers through an integrated approach will be essential to improving HPV vaccine uptake, ensuring equitable protection for eligible girls, and ultimately reducing the burden of cervical cancer in Nigeria. To provide a structured and actionable summary of these findings, Table A1 aligns the identified implementation challenges with context-appropriate recommendations.

## Limitations

This study has limitations that should be considered when interpreting the findings. First, it was conducted in three Nigerian states with purposive sampling; while Kaduna, Kano, and Lagos are diverse, the findings may not reflect all regions or socio-economic contexts in Nigeria. Second, the analysis draws on rapid synthesis of qualitative data; a more in-depth coding and triangulation may yield additional insights. Finally, data rely on self-reports and recollections, which are subject to recall and social desirability biases. Further study limitation includes limited inclusion of caregivers/adolescents, and the absence of direct observation of service delivery.
